# RatHat: A Self-Targeting Printable Brain Implant System

**DOI:** 10.1523/ENEURO.0538-19.2020

**Published:** 2020-03-31

**Authors:** Leila M. Allen, Maanasa Jayachandran, Tatiana D. Viena, Meifung Su, Bruce L. McNaughton, Timothy A. Allen

**Affiliations:** 1 Cognitive Neuroscience Program, Department of Psychology, Florida International University, Miami, FL 33199; 2 Department of Neurobiology and Behavior, University of California, Irvine, CA 92697; 3 Department of Environmental Health Sciences, Robert Stempel College of Public Health, Florida International University, Miami, FL 33199

**Keywords:** cannula, electrodes, optrodes, neurosurgery, rodent surgery, stereotaxic, tetrodes

## Abstract

There has not been a major change in how neuroscientists approach stereotaxic methods in decades. Here, we present a new stereotaxic method that provides an alternative approach to a traditional u-frame stereotaxic device and reduces costs, surgical time, and aids repeatability. The RatHat brain implantation system is a 3D-printable stereotaxic device for rats that is fabricated prior to surgery and fits to the shape of the skull. RatHat builds are directly implanted into the brain without the need for head-leveling or coordinate-mapping during surgery. The RatHat can be used in conjunction with the traditional u-frame stereotaxic device, but does not require the use of a micromanipulator for successful implantations. Each RatHat contains several primary components including the implant for mounting intracranial components, the surgical stencil for targeting drill sites, and the protective cap for preventing damage from impacts and debris. Each component serves a unique function and can be used together or separately. We demonstrate the feasibility of the RatHat in four different proof-of-principle experiments: (1) a three-pole cannula apparatus, (2) an optrode-electrode assembly, (3) a fixed-electrode array, and (4) a tetrode hyperdrive. Implants were successful, durable, and long-lasting (up to nine months). RatHat print files are easily created, can be modified in computer aided design (CAD) software for a variety of applications, and are easily shared, contributing to open science goals and replications. The RatHat has been adapted to multiple experimental paradigms in our lab and should be a useful new way to conduct stereotaxic implant surgeries in rodents.

## Visual Abstract

**Figure:**
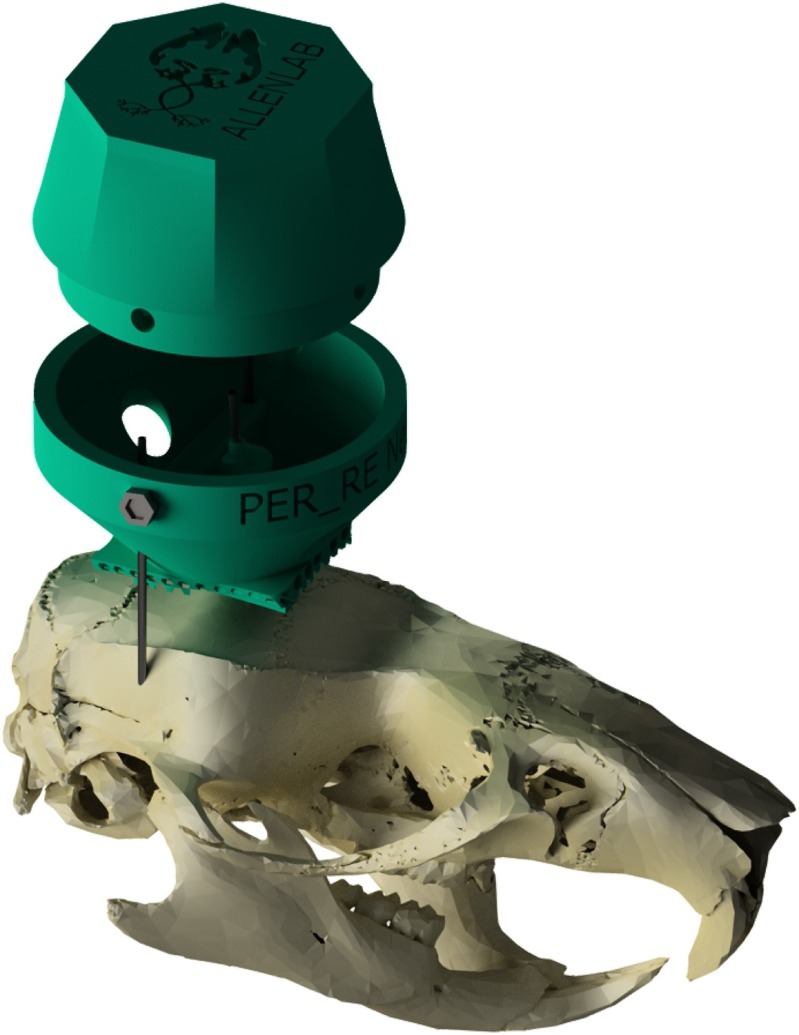

## Significance Statement

We demonstrate a new approach to rodent stereotaxic surgery. Rodent neurosurgery is a complex skill that requires expensive equipment for head stabilization and micromanipulators for localization. The RatHat is a 3D-printable brain implant system that reduces costs and time using pre-mapped and printed surgical files. A surgical stencil allows for quick placement of drill holes, and a RatHat places components in the brain using atlas coordinates. The RatHat is an easily shared resource facilitating open science goals for replications and the archiving of specific experimental applications.

## Introduction

Rodent neurosurgery is challenging to master, especially for surgeries involving multiple target sites. Long surgeries cause surgeon fatigue and distress in animals, affecting recovery time and surgical outcomes (Hoogstraten-Miller and Brown, 2008; Pritchett-Corning et al., 2011; Ferry et al., 2014; Fox, et al., 2015). With the increased emphasis on circuit analysis and multiple targets (e.g., DREADDs and optogenetics), opportunities for positioning errors are increased (Jorgenson et al., 2015; Bassett and Sporns, 2017; Jayachandran et al., 2019).

Typically, brain implants are placed using a u-framed stereotaxic apparatus in which the rat’s head is stabilized with ear and tooth bars, putting the rat into 3D atlas space (Paxinos and Watson, 2013). Micromanipulators allow implants to be precisely moved in xyz coordinate planes. However, this setup can introduce unrecoverable user errors that go unnoticed. For example, while surgeons are trained to level the head in the anterior/posterior (A/P) plane, many fail to level in the medial/lateral (M/L) plane yielding asymmetrical implants/injections that unnecessarily increase the number of animals needed (Fornari et al., 2012; JoVE Science Education Database, 2019). Notably, there has not been a major change in how neuroscientists approach stereotaxic methods in decades.

As a practical issue, a standard u-frame surgical apparatus can range from $5000 to $50,000 (or more with addition of specialty add-ons), costing research labs a considerable portion of their budgets and presenting a bar-to-entry for less well-funded laboratories. In relation, an adequate high-resolution 3D printer currently costs around $3k new (e.g., 3D Systems, FabPro 1000), and many universities and institutions have shared printers which can be used for a nominal materials fee (e.g., $10/h at Florida International University).

Here, we introduce a customizable, fully integrated 3D-printable stereotaxic brain implant system called RatHat that is freely available to academic researchers (Allen et al., 2017, 2019a). The RatHat can be used in conjunction with, or as an alternative to the u-framed stereotaxic apparatus for methods requiring atlas-based positioning. A key feature is that the system self-aligns to atlas space because it fits the skull, eliminating the need for micromanipulator measurements and head leveling.

The RatHat has reduced costs in our lab compared with commercially-available equivalents, and has reduced surgery time (Table 1). It is customizable for a variety of surgical applications through computer aided design (CAD) modifications prior to surgery (e.g., Autodesk, Blender, etc.). RatHat files are easily shared over the Internet and archived for later use with versioning (aiding new experiments and replications). Printouts are considerably less expensive than similar commercial products while providing a larger range of possibilities (Table 1).

**Table 1:** Time and cost of using RatHat

Sample time and cost for using RatHat^a^				
	**Cannula RatHat**	**Optrode RatHat**	**Single wire RatHat**	**Tetrode RatHat**
Print time (in-house)^b^	2 h	4 h	2 h	3 h 30 min
Materials (not including glue)	3D prints, cannula, sand paper,^c^ dummy cannula	3D print, EIB,^c^ stainless-steel wire, gold pins, optrode	3D print, EIB,^c^ stainless-steel wire, gold pins	3D print, EIB,^c^ gold pins, tetrode screws,^c^ support rods,^c^ tetrodes
Assembly time	15 min	50 min	1 h 30 min	8 h
Surgery time	1 h 30 min	2 h 20 min	2 h	2 h 30 min
Cost of printing in-house	$1.25	$4.50	$3.25	$6.25
Cost of university printing ($10/h)	$20.00	$40.00	$20.00	$35.00
Cost of 3D printing service	$35.66	$44.92	$35.29	$32.15
Cost of other materials	$2.23	$163.82	$153.40	$266.70
Equivalent quoted commercial cost	$81.00 (3 individual cannula) $161.66 (bilateral cannula setup plus one individual cannula)	No equivalent commercial product available	$330.00	$1,870.00 (assembly required and does not include tetrodes)

a Times and costs listed are an approximation from data obtained in our lab (http://allenlab.fiu.edu/).

b Print times are the sum of printing all necessary parts.

c Reusable material.

RatHat applications have been adopted for a variety of experimental needs. Here, we demonstrate the use of RatHat in four experimental applications: multisite chronic cannula, multisite optrode-electrode implants, a microwire microarray, and a tetrode hyperdrive.

RatHat is freely available to academic researchers, achieving open science goals. Academic researchers interested in receiving the 3D files can contact Dr. Timothy A. Allen. We will first provide you a license to be executed by your institution, and on completion, 3D files of the implant system.

## Materials and Methods

RatHat components were printed using the 3DSystems ProJet1200, a high-resolution (56-μm *xy*, 30-μm layer thickness) 3D printer that uses microstereolithography (laser polymerization of resin and UV light-curing), but any high-resolution 3D printer is suitable. With ProJet1200 prints, we use VisiJet FTXGreen resin, a UV-curable and biocompatible plastic composition used in castings because it is durable, with a tensile strength of 30 MPa (or 4351 PSI). After devices are printed, we ensure holes are clear of debris or resin by thoroughly cleaning prints with multiple dips in 70% isopropyl alcohol and clearing holes with pressurized air. Non-printable components such as wires or tubing are secured to the implant device prior to surgery with cyanoacrylate (Zap CA+, Super Glue Corporation) followed by a quick-cure spray (Zip Kicker, Super Glue Corporation). Another advantage of the RatHat is that components are easily assembled using build-specific 3D-printable assembly bases/jigs. All implants are sterilized with 70% ethanol before surgical implantation and a gas sterilizer (ethylene oxide). Autoclaving is not recommended.

### Components: RatHat implant, surgical stencil, protective cap, and implant jig

Several components are common to all designs. The RatHat is a stable and secure housing apparatus for long-term neurosurgical implants. It is secured to the skull with anchor screws and dental cement. The RatHat underside contains horizontal channels for dental cement designed to optimize long-term adhesion to the skull and anchor screws (up to nine months in our experiments). Identifying information about the animal/experiment can be included on the print as well.

The surgical stencil contains all alignment and drill holes for the target sites needed in the surgery and was designed to facilitate rapid and accurate drilling of implantation and/or infusion sites matching the RatHat implant base. The surgical stencil is a transformative device for any surgeon to rapidly and cleanly introduce holes or craniotomies. It is easy to print and uses relatively small amounts of resin, so multiple copies can be used for a single surgery in case a back-up is needed. This also helps with making straight and unbiased holes during free-handed drilling.

The protective cap safeguards other RatHat components (e.g., cannula, dummies, electrodes, drives, etc.) from dust, debris, and impacts. It mounts on the RatHat implant sidewalls and is secured with a screw. The walls and the protective cap are outfitted with screw-holes for alignment on all sides to accommodate both left-handed and right-handed surgeons. The protective cap can be printed with individualized lab insignia and/or animal names for identification. Protective caps can be easily replaced with a reprint.

The jig serves to model the brain space and allows for precise placement and securing of implant components such as cannula tubes in the RatHat prior to surgery. In order to prepare RatHat cannula implants for surgery, the cured and cleaned 3D print is placed inside the jig. Next, pre-measured and cut stainless-steel tubes (27 gauge, Component Supply Company) are placed into the RatHat through corresponding holes in the jig. The D/V depths of the cannula are dictated by CAD-measured ledges printed within the jig. This reduces fabrication time and more importantly, measurement errors. Once the stainless-steel cannulae are secured to the RatHat, the device can be implanted in the brain without the need for coordinate mapping during surgery. In this way, the jig replaces the dorsoventral (D/V) component of a stereotax micromanipulator arm, allowing for hand implants (no u-framed stereotaxic device) if comfortable doing so.

### Animals and general surgical methods

Subjects were Long–Evans rats that weighed 250–275 g on arrival (*n* = 26, 2 females). All rats included were used in other primary experiments. Rats were individually housed in clear polycarbonate cages to ensure implants were protected from damage by cage-mates. Rats were maintained on a 12/12 h light/dark cycle (lights off at 10:00 A.M.). Naïve rats were briefly handled for 3–5 d after arrival. Access to food and water was unrestricted before surgery. All surgical and behavioral methods were in compliance with the Florida International University Institutional Animal Care and Use Committee (IACUC) and Institutional Biosafety Committee (IBC).

**Figure 1.**
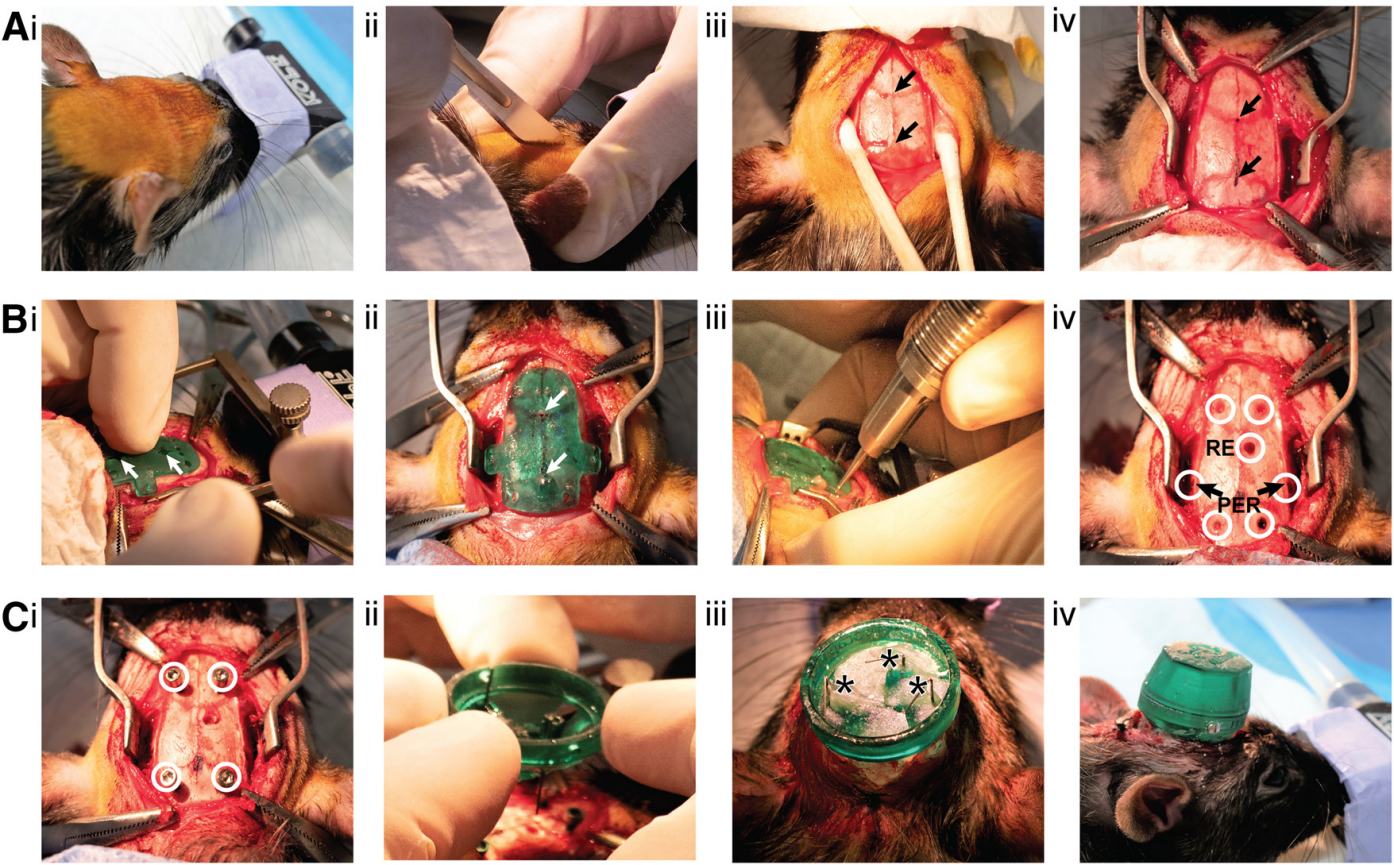
RatHat surgical procedures. ***A***, Prep: i, Prepare the skin for incision. ii, Make the incision. iii, Clean skull and expose bregma and lamda. iv, Mark bregma and lamda and secure clamps to periosteum as needed. ***B***, Drill: i, Place the stencil on the skull and align holes to bregma and lamda, indicated by white arrows. ii, Glue the stencil to the skull using cyanoacrylate and a quick-cure spray. iii, Drill holes according to the stencil. iv, Holes for skull screws and RE and PER cannula shown (white circles indicate drill sites for both cannula and skull screws). ***C***, Implant: i, Insert skull screws (white circles indicate inserted skull screws). ii, Manual placement of the preassembled cannula RatHat implant base. iii, Dental cement RatHat to the skull and insert dummies (asterisks indicate location of cannula poles). iv, Place the protective cap and secure with a screw. For surgical procedure also see https://youtu.be/W9zV6lIoIus. RE, nucleus reuniens of the thalamus; PER, perirhinal cortex.

Surgically implanting the RatHat follows basic techniques for intracranial survival surgery (Fig. 1). Briefly, general anesthesia was induced (5%) and maintained by isoflurane (1–2.5%) mixed with oxygen (800 ml/min). Rats were placed in a stereotaxic apparatus in the sterile surgical field for stabilization with ear-bars and tooth-bars (although RatHat surgeries can be performed without bars). Rats were administered glycopyrrulate (0.2 mg/ml, 0.5 mg/kg, s.c.) and 5 ml Ringer’s solution with 5% dextrose (subcutaneously) for hydration. Temperature was monitored with a rectal thermometer and maintained within ±1C° of baseline with a heating pad. The skull was exposed following a midline incision or fish-eye cut (Fig. 1*A*). The periosteum was detached from the skull using cotton-tipped applicators (Puritan Medical Products) and clamped with hemostats to expose the width of the skull up to the lateral ridges (and 2 mm beyond the ridges when accessing more lateral structures) and 3–4 mm in length (A/P) beyond bregma and lambda. Score marks were made on the skull using the scalpel blade for dental cement adhesion.

The surgical stencil was aligned to bregma and lambda using landmark holes that are surrounded by crosshairs to facilitate visualization and placement (Fig. 1*B*). The stencil was secured to the skull using cyanoacrylate (Zap CA+, Super Glue Corporation) followed by a quick-cure spray (Zip Kicker, Super Glue Corporation). Drill holes were made in appropriate regions according to the specific RatHat build using a drill (OmniDrill 3S, World Precision Instruments). Dura mater was ruptured at the implant sites using a 32-Gauge needle. The stencil was removed using a scalpel blade or a spatula and discarded. Excess cyanoacrylate was scraped off the skull to clear debris that could interfere with placement of the RatHat implant. The skull was thoroughly cleaned with sterile saline or hydrogen peroxide (avoiding contact with soft tissue) to ensure successful long-term adherence of the RatHat. Next, titanium anchor screws were secured (Fig. 1*C*). The RatHat was aligned to drill holes and carefully lowered into place using a micromanipulator arm or by hand fitting it flush with the skull. Dental cement was applied in layers to secure the base to the anchor screws and skull using a wooden applicator tip, a syringe, or a paint brush (saturated in the curing liquid and then used to pick up dry powder which polymerized into the cement and facilitated a smooth and well-anchored implant free of jagged edges). The inside of the implant was filled with dental cement to further stabilize components. The protective cap was secured onto the wall of the RatHat using a small screw. The posterior incision was sutured if necessary, rats were administered an analgesic (flunixin, 50 mg/ml, 2.5 mg/kg, s.c.), and topical antibiotic ointment was applied. The rat was placed in a postsurgical recovery incubator until awake and moving, and then returned to a clean home cage. A day following surgery, rats were given an analgesic (flunixin, 50 mg/ml, 2.5 mg/kg, s.c.) and topical antibiotic ointment was applied. The protective cap was removed to check that the RatHat implant components were in good condition. Rats were monitored postoperatively for a week and then resumed experimental testing.

Upon completion of the experiments, intracranial placements were mapped using postmortem brain slices. Rats were induced under general anesthesia using isoflurane (5%) and transcardially perfused with 100 ml of ice-cold 0.1 M PBS followed by 200 ml of 4% paraformaldehyde (pH 7.4; Sigma-Aldrich). Brains were postfixed overnight in 4% paraformaldehyde and then cryoprotected in a 30% sucrose and 0.1 M PBS solution prior to sectioning (Leica CM3050S, Leica Biosystems). Three sets of immediately adjacent sections (40 μm, coronal) were saved. One set was mounted onto glass slides for Cresyl Violet staining.

## Results

### Experiment 1: Three-pole cannula RatHat for simultaneous implantation of multiple cannula (Fig. 2)

Commercially-available multisite cannula assemblies from vendors such as inVivo1 and WPI are custom ordered, requiring a necessary lead-time, and very expensive [e.g., ∼$20–30 per individual cannula, including guides and dummies (WPI); $340 for 10 bilateral cannula assemblies (inVivo1); Table 1]. Furthermore, they only accommodate up to two cannulas anchored together by a thin plastic tether, and are unable to incorporate poles for angled insertions.

**Figure 2.**
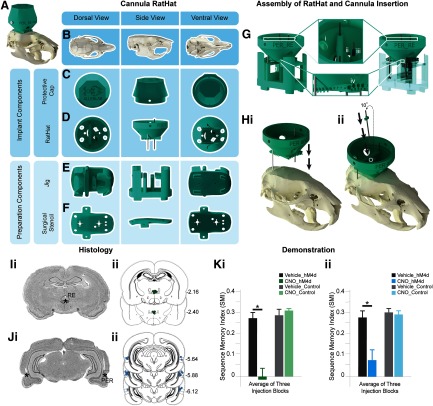
Three-pole cannula RatHat. ***A***, Full RatHat cannula assembly on the skull. ***B***, Rat skull in different orientations. ***C***, Protective cap shown in different orientations. ***D***, RatHat implant base with preassembled cannula. ***E***, The jig is used to assemble the cannula tubes into the RatHat implant prior to surgery. ***F***, The stencil contains reference marks for bregma and lamda to align to the skull; once adhered, all drill marks are properly placed for cannula access points and anchor screws, saving time. The stencil is removed and discarded after drill holes are made. ***G***, The cannula RatHat sitting on top of the jig. The star indicates where the cannula stops in order to glue the cannula directly to the RatHat or glue the depth stop to the cannula at the correct DV. i, Anchor screw holes are large so they do not obstruct the RatHat’s fit-to-skull. ii, Dental cement application holes. iii, The depth stop on the angled cannula allows to place the cannula by hand after the implant base is secured on the skull, while ensuring it descends to the correct DV. iv, Channels on the underside of the RatHat increase surface area, facilitating dental cement adhesion to ensure a strong long-term bond between the RatHat Implant and skull. ***H***, i, The RatHat assembly (including bilateral PER cannula) is lowered onto the skull first. ii, Then the RE cannula with a pre-glued depth stop is inserted (the white circle indicates the RE hole). ***I***, i, Sample coronal slice. The asterisk indicates the infusion cannula tip location in RE. ii, Microinfusion injector tip location in the RE for all rats (*n* = 13). Numbers to the right of each section indicate distance (mm) anterior to bregma. ***J***, i, Sample coronal slice. The asterisks indicate the infusion cannula tip location in PER. ii, Microinfusion injector tip location in the PER for all rats (*n* = 13). Numbers to the right of each section indicate distance (mm) anterior to bregma. ***K***, Rats were injected with AAV-hM4Di (an inhibitory DREADD) in mPFC or a control virus, and a cannula targeted RE and PER (bilaterally). Well-trained rats were infused with CNO in RE and PER (the DREADD agonist) or vehicle prior to testing. i, Silencing the mPFC → RE terminals (the CNO-hM4Di group) significantly impaired sequence memory (dependent *t*_(9)_ = 7.074, *p *=* *0.0409). ii, Silencing the mPFC → PER terminals (the CNO-hM4Di group) significantly impaired sequence memory (dependent *t*_(9)_ = 0.2005, *p *=* *1 × 10^−4^). CNO, clozapine N-oxide; mPFC, medial prefrontal cortex; RE, nucleus reuniens of the thalamus; PER, perirhinal cortex. The data were sourced from Jayachandran et al. (2019).

The RatHat cannula system contains multiple pre-measured cannulas assembled before surgery, reducing surgical time by eliminating the need to identify coordinates with micromanipulators and make insertions one-at-a-time. Here, two cannulas targeted perirhinal cortex (PER) bilaterally, and one cannula targeted the nucleus reuniens of the thalamus (RE). PER cannulas were affixed to the RatHat prior to surgery using the jig, while the RE cannula was placed during surgery after the RatHat was secured to the skull (Fig. 2*G*,*H*). PER is a good site to demonstrate the RatHat cannula approach because it is a difficult structure to access, given its depth and laterality (A/P, −3.0 to −7.0; M/L, ±0.7.2; D/V, −6.5 to −7.5; Burwell, 2001; Paxinos and Watson, 2013). The third cannula targeted RE, a structure that lies directly below the superior sagittal sinus (SSS; A/P, −1.08 to −3.48; M/L, ±0.08; D/V, −6.8 to –7.8). SSS can easily rupture, prolonging surgical time and causing significant damage or death. Thus, we incorporated an angled cannula pole (10°) into this RatHat design to target RE and avoid SSS. This angled pole is fitted with a depth-stop, eliminating the need for D/V measurements, and was inserted by hand into the RatHat (Fig. 2*Hii*).

Rats (*n* = 13) were trained in an odor sequence memory task (Jayachandran et al., 2019) followed by RatHat implantation surgery. We compared surgery times using the RatHat (*n* = 13, surgery time: 104.000 ± 4.785 min) to a traditional u-frame stereotaxic approach (*n* = 5, average surgery time: 183.800 ± 13.002 min). Surgeries conducted using the RatHat took significantly less time than the traditional u-frame stereotaxic approach (*t*_(4)_ = 6.534, *p *=* *0.003). Once recovered, they resumed behavioral testing which demonstrates the durability of the RatHat, an ideal device for experiments that require extended testing periods and involve extensive task related wear-and-tear. These rats completed ∼60 sessions after surgery, with 200–300 nose-pokes/session. Additionally, rats were given 12 infusions over several weeks to either PER (bilaterally) or RE. Infusions targeted the structures of interest and resulted in distinct sequence memory disruptions that relate to the functioning of those regions. RatHat implants stayed on for an average of six to nine months when rats were euthanized for histologic analysis. Overall the accuracy (measured by distance from the intended target) and reliability (consistently hitting the same spot, measured by distance from the observed centroid) of RatHat cannula placements were not significantly different from the traditional u-frame stereotaxic approach (RatHat_TargetDistance_: RE, 0.294 ± 0.096 mm and PER, 0.320 ± 0.121 mm; RatHat_CentroidDistance_: RE, 0.246 ± 0.135 mm and PER, 0.254 ± 0.101 mm; TargetDistance_RatHat versus Traditional_: RE, *t*_(4)_ = −2.329, *p *=* *0.080 and PER, *t*_(9)_ = −1.069, *p *=* *0.313; CentroidDistance_RatHat versus Traditional_: RE, *t*_(4)_ = −1.180, *p *=* *0.304 and PER, *t*_(9)_ = −0.431, *p *=* *0.676; Fig. 2*I*,*J*). Notably, we have not seen any major differences in entry damage to the brain when using the RatHat compared with the traditional u-frame stereotaxic approach.

### Experiment 2: RatHat design for a combination of optogenetics and microwire recordings (Fig. 3)

We implanted rats (*n* = 4; 2 females) weighing ∼275–350 g at surgery. The optrode targeted the junction of the RE body and RE wing (perireuniens; A/P, −2.3; M/L, −0.5; D/V, −7.0) and was implanted vertically slightly lateral to the midline (a different approach compared with the cannula; Jayachandran et al., 2019). After the holes were drilled, an injection of AAVr-CAG-hChR2-H134R-tdTomato (channelrhodopsin virus; Addgene catalog #28017) or pAAVr-CAG-tdTomato (control virus; Addgene catalog #59462) was made using pulled glass pipettes (P-2000 Laser-Based Micropipette Puller, Sutter Instruments) with a tip diameter between 80 and 100 μm driven by a motorized infusion pump (0.3–0.5 μl at 60 nl/min; Nanoject III, Drummund Scientific). Because the optrode has a built-in depth-stop for the D/V axis, a jig was not required for this version.

**Figure 3.**
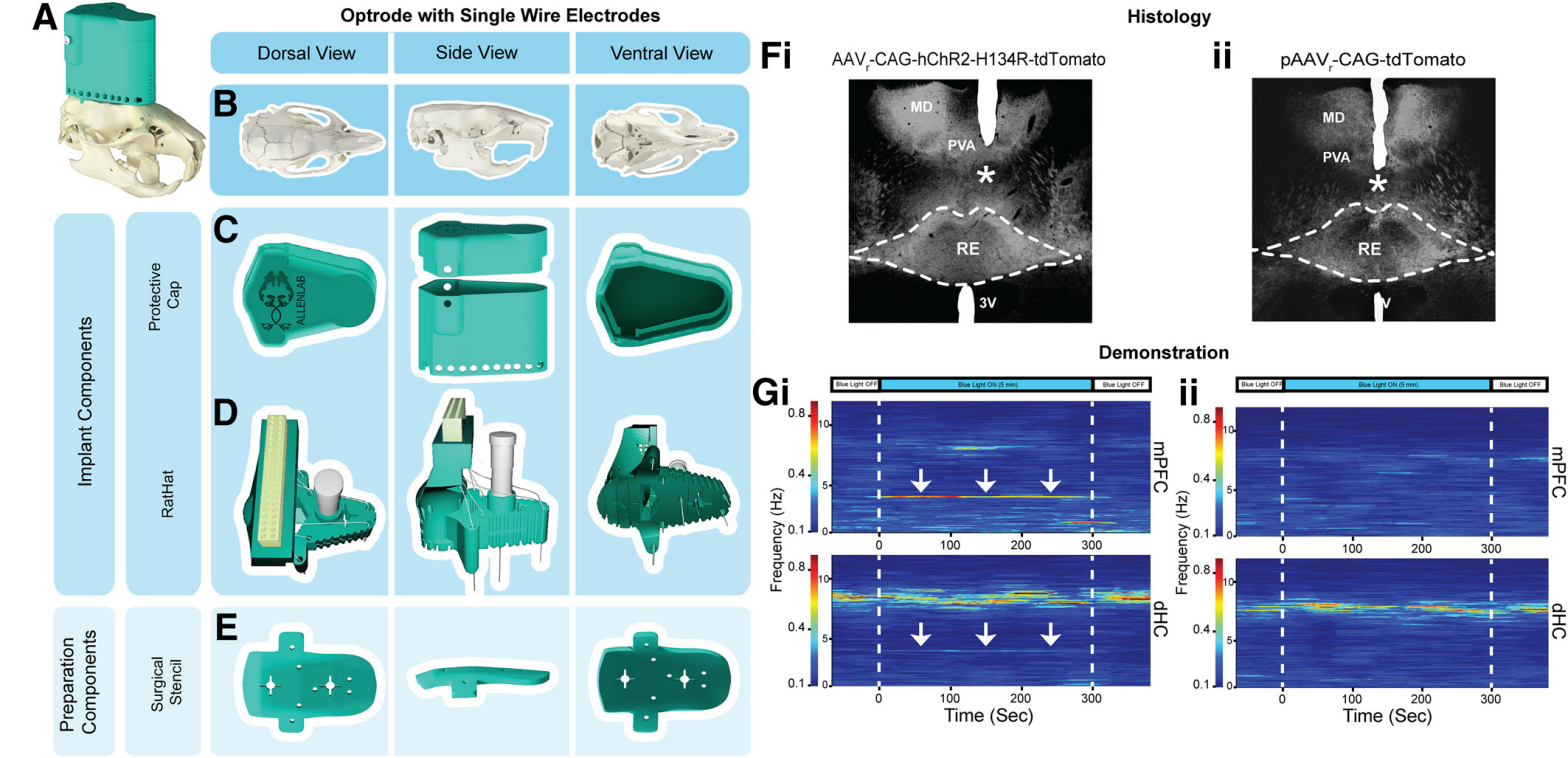
Optrode and SS wire electrode RatHat assembly. ***A***, Optrode/electrode RatHat implant on an average sized rat skull. ***B***, Rat skull viewed across different anatomic planes. ***C***, View of the RatHat protective cap and wall in different orientations. These items protect the internal components post implantation. ***D***, RatHat implant base (in teal) preassembled with optrode and electrode single wires. ***E***, RatHat surgical stencil showing prefabricated holes that correspond to brain coordinates of interest, bregma and lamda, and screw locations for rapid drilling on the skull. ***F***, Brain sections showing channelrhodopsin (i) and viral control (ii) expressed neurons in the midline thalamus and the optrode placement (asterisk) just above RE in representative cases, demonstrating the effectiveness of using the RatHat. ***G***, Perievent spectrograms of representative mPFC and dHC LFP showing the 5-min period in which the blue LED light was administered via the optrode (see asterisk for tip location) activating ChR2 ion channels in infected (i; AAVr-CAG-hChR2-H134R-tdTomato) and control (ii; pAAVr-CAG-tdTomato) rats. Also shown, 60 s before and after the stimulation block. Pulsed blue light activation (4 Hz, 60-ms pulse width) of RE ChR2+ neurons elicited a 4-Hz frequency rhythm (see arrows) in the mPFC (strong) and dHC (weak) LFP signal. We also observed comparable frequency-specific activations at 1, 2, and 8 Hz. This change, however, was not observed in control animals (on right; Viena et al., 2019). mPFC, medial prefrontal cortex; dHC, dorsal hippocampus; RE, nucleus reuniens of the thalamus; PVA, paraventricular nucleus; MD, medial dorsal nucleus; ChR2, channelrhodopsin.

We `show sample data in which optogenetic stimulation of RE in experimental rats yielded a 4-Hz frequency rhythm in the mPFC (strong) and dHC (weak) LFP signal, but not in controls, demonstrating efficacy of the optogenetic approach (Viena et al., 2019; Fig. 3*G*). Implants remained in place for 4.5 months until histologic analysis. Proper placement of the optrode and electrode wires was verified and consistent in all rats (Fig. 3*F*).

### Experiment 3: RatHat for implanting fixed stainless-steel wire arrays

**Figure 4.**
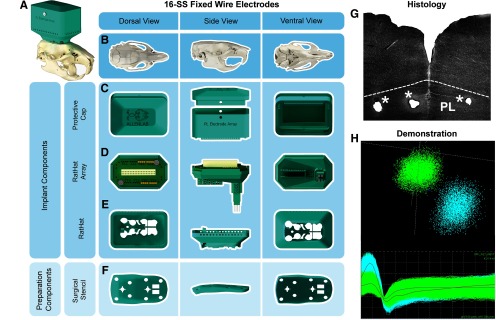
Sixteen single wire fixed electrode array RatHat. ***A***, Fixed electrode array RatHat on the skull. ***B***, Rat skull in different orientations. ***C***, The protective cap and wall that protects the electrode array after implantation. ***D***, The RatHat electrode array with preassembled fixed stainless-steel single wires docks into the RatHat implant base. ***E***, The RatHat implant base is anchored to the skull before the RatHat electrode array is docked, ensuring the wires descend to the correct DV. ***F***, The stencil contains reference marks to align bregma, as well as a craniotomy window and drill holes for anchor and ground screws. ***G***, Sample coronal slice of the 16-wire SS electrode array RatHat. The asterisks indicate single wire tip locations. ***H***, Sample cluster plot showing two isolated mPFC units on a single channel during free-roaming behavior. PL, prelimbic cortex.

Targeting prelimbic (PL) and infralimbic (IL) regions of the medial prefrontal cortex (mPFC; Fig. 4) were piloted for feasibility in male rats (*n* = 2; ∼350 g at surgery). The electrode array was built similar to those used in other experiments (Krupa et al., 2004; Narayanan and Laubach, 2006). The surgical stencil for this version included a craniotomy window supporting electrode arrays bilaterally targeting PL/IL (A/P, 1.7–4.0; M/L, ±1.8; D/V, −3.0) in addition to landmark and anchor screw holes. Once the craniotomy and drill holes were made, anchor screws were inserted. Next, the RatHat implant was secured to the skull. The electrode array was then inserted into the brain, docked into place on the RatHat, and secured with dental cement. The protective wall was secured with dental cement (filling in the base up to the electrode interface board). Once dry, we plugged the rat into the electrophysiological recording system (Plexon) to assess neural activity. Afterwards, the protective cap was secured. Neural activity was measured over the course of the next several months. RatHat electrode arrays remained in place for approximately four months with good signal. We successfully recorded well-isolated single-unit activity (Fig. 4*H*). Marking lesions were performed using a NanoZ for localizing electrode sites (Fig. 4*G*).

### Experiment 4: Eight-tetrode hyperdrive RatHat (Fig. 5)

**Figure 5.**
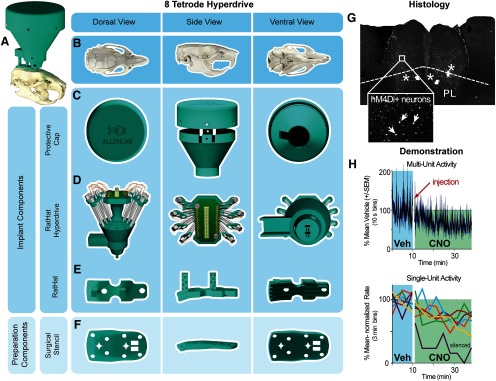
Eight-wire tetrode hyperdrive RatHat. ***A***, The fully assembled eight-wire tetrode hyperdrive RatHat on the skull. ***B***, Rat skull in different orientations. ***C***, The protective cap and wall ensure the RatHat hyperdrive is safe from impacts and debris. ***D***, The hyperdrive with preassembled drivable tetrodes targeting regions in mPFC. ***E***, The RatHat implant base is secured to the skull and has docking poles on which the RatHat hyperdrive sits, ensuring the tetrode tips are placed right above cortex. ***F***, The stencil aligns to bregma and lambda and contains guide holes for drilling craniotomies and anchor screw holes. ***G***, Sample slice with eight-wire tetrode hyperdrive RatHat. Asterisks indicate the tetrode wire tips. ***H***, Implanted tetrodes in mPFC with hM4Di expression showing functional inhibition following CNO injection (1 mg/kg). CNO, clozapine N-oxide; mPFC, medial prefrontal cortex; PL, prelimbic cortex; Veh, vehicle.

Microdrive screws and shuttles for the RatHat hyperdrive were assembled and implanted similar to others (Wilson and McNaughton, 1993; Gray, et al., 1995; Nguyen, et al., 2009). The tetrode array was securely encased in the protective wall prior to surgery. We implanted the RatHat hyperdrive in male rats (*n* = 2; ∼350 g at surgery). This stencil version was the same as that used in experiment 3. After the RatHat implant base was secured to the skull, the RatHat hyperdrive was carefully placed by hand and secured onto the base with dental cement. Immediately after, tetrodes were driven 1 mm, and the rat was plugged into the electrophysiological recording system (Plexon) to assess signal. Once functionality was established, the rat was unplugged and the protective cap was secured. Tetrodes were driven 250 μm/d until reaching a depth of 2.8–3.0 mm (D/V; staggered) with a goal of recording mPFC cells (A/P range, 4.7–2.5; M/L range, ±0.2 to ±1.6). The hyperdrive successfully isolated single-units in mPFC of freely-behaving rats, demonstrating the RatHat application (Fig. 5*H*). Four weeks after implantation, marking lesions were made for histologic verification (Fig. 5*G*).

## Discussion

RatHat is a 3D-printed stereotaxic device that can be used for a range of applications, such as cannula placements, microinfusions, optogenetics, and electrophysiological recordings. The RatHat was developed to reduce surgical time while providing accuracy and reliability, and to contribute to open science goals. This is a major change to current stereotaxic approaches because we replaced an approach that has been employed for several decades that uses micromanipulators for measurements during surgery. The RatHat saves time, money, and offers reliability. In addition, the build files for any specific implant can be easily shared between researchers and labs facilitating experimental replications using identical approaches. The fundamental system consists of complementary components including a RatHat implant base, a surgical stencil, a jig, and a protective cap. Here we demonstrated four different RatHat systems for feasibility in multiple types of neuroscience experiments. We verified the durability of these implants, which remained in place for up to nine months, despite movement-rich and impact-dense behavioral tasks (Jayachandran et al., 2019).

We plan to develop a RatHat for other commonly used species in neuroscience, including mice. We have also developed a version for chronic implants in the domestic pig, which facilitates surgery without the need for a traditional large-animal stereotaxic apparatus (HogHat; Allen et al., 2019b). In addition, RatHat versions for other common neurosurgical applications are underway including an acute implant device that allows for single injections of excitotoxins, AAVs, DREADDs, etc.

Again, we make the RatHat freely available for all academic researchers to aid in their experiments and contribute to open science goals. We look forward to seeing new builds and implementations.

## Synthesis

Reviewing Editor: Mark Laubach, American University Decisions are customarily a result of the Reviewing Editor and the peer reviewers coming together and discussing their recommendations until a consensus is reached. When revisions are invited, a fact-based synthesis statement explaining their decision and outlining what is needed to prepare a revision will be listed below. The following reviewer(s) agreed to reveal their identity: Benjamin Clark.

Comments from Reviewer 1:

The manuscript presents an exciting new methodological development - a 3D printable stereotaxic device that could reduce costs and surgical error associated with common behavioral neuroscience applications. The manuscript is very well written and the feasibility of the design is clearly presented and justified. Below I list a few minor comments regarding presentation and description.

Comments from Reviewer 2:

Manuscript #eN-OTM-0538-19 describes a novel, alternative approach to current rodent stereotaxic neurosurgery aptly named RatHat. A surgical stencil basically provides the surgeon with a layout of the locations where holes need to be drilled after lining up the stencil to bregma and lambda, both conveniently marked with crosses in the stencil, and can be modified for each surgery depending the experimental needs. The stencil is then removed and the RatHat base is attached to the skull, which houses the to-be-implanted items such as cannula, electrodes or optic fibers.

The system conveniently utilizes 3D printed components that can be modified depending on the requirements of the experiment and potentially allows the user to forego an expensive stereotaxic frame. The development and dissemination of such novel systems is critically important to the continued success of neuroscience and I believe the RatHat system has immense potential to further our neuroscientific pursuits. Ultimately I think the claims made in the manuscript go beyond what is actually presented. It would be great if the authors were able to justify the claims highlighted below, but the RatHat system has great value even as an alternative to standard surgical approaches even if it is not conclusively superior.

Synthesis of reviews (Essential Revisions)

1) In both the Introduction (line 51) and Discussion (lines 239-243) a number of claims are made regarding the RatHat system, which do not seem to be supported by anything presented in the manuscript.

• Line 51: “The RatHat system reduces costs, training, and surgery time.”

• Lines 238-240: “The RatHat system was developed to improve surgical accuracy and precision, reduce surgery time in rodent neurosurgical procedures, and contribute to open science goals.”

• Lines 243-244: “The RatHat system saves time, money, offers reliability, and provides for surgical replications.”

- There does not seem to be any data presented showing that the RatHat system improves surgical accuracy/precision. Representative examples are shown that targets are appropriately reached, but that is the case with current standard stereotaxic neurosurgical approaches as well.

- There does not seem to be anything presented about the duration of surgery when using the RatHat system, particularly in comparison to standard stereotaxic neurosurgical procedures.

- There does not seem to be anything presented about the amount of training required to learn the RatHat system, particularly in comparison to standard stereotaxis neurosurgical procedures.

- There does not seem to be anything presented about how much the RatHat system costs to implement. How much does a 3D Systems Projet 1200 cost? Or how much would it cost to get parts printed commercially instead of buying one’s own 3D printer?

All of these points are simply trying to get at the issue that the manuscript does not appear to provide justification for claiming that the RatHat system is superior to standard stereotaxic surgery. RatHat is clearly an alternative system, but it is not clear where it stands relative to the current standard procedures.

- Please revise the manuscript to address these points or remove claims about cost, training, and surgery time.

2) Please add more details about the time and cost associated with each RatHat application, i.e. how long it takes to assemble the RatHat and perform surgeries for each experiment. This should be mentioned in the text or in a table.

3) Please clarify in the description of Experiment 1 how the cannula are implanted. Are they embedded in the RatHat base and therefore lowered into the brain when the RatHat base is lowered onto the skull? Or is the base attached to the skull and then the cannula are inserted to pre-measured holes with some sort of stopping mechanism in order to ensure they reach the appropriate D/V depth?

4) In the section describing the surgical procedures, please add a reference for Figure 1.

5) In Figure 1, please add labels for the PER and RE targets. Also, what do the white circles indicate? The drill sites? This should be mentioned in the caption.

6) In Figure 2, an issue was raised about how the jig is used to load cannula into the RatHat. Would it be possible for the authors to provide some diagrams or photos to illustrate this?

7) In Figure 2, the asterisk marking significance should be defined and statistical methods should be described briefly in the text. If this data is adapted from a previous study, the work should be cited in the caption or in text.

8) Line 179-182: Could an illustration be added to show the surgical targets and modified device, “angled cannula pole (10{degree sign})”?

9) Line 192 - Is the optrode design an adaption from a previous study? If so, the authors should cite the previous work here.

10) Please add a comment about the potential damage to brain tissue when implants done are by hand compared to using a micromanipulator attached to a stereotaxic frame.

11) The manuscript mentions an interactive 3D environment for mapping out coordinates (line 56), yet this is never mentioned again in the manuscript. If this is a component of the RatHat system, then it should be described in the manuscript or the comment should be removed from the manuscript.
